# Prevention of inflammation-mediated acquisition of metastatic properties of benign mouse fibrosarcoma cells by administration of an orally available superoxide dismutase

**DOI:** 10.1038/sj.bjc.6603016

**Published:** 2006-02-28

**Authors:** F Okada, H Shionoya, M Kobayashi, T Kobayashi, H Tazawa, K Onuma, Y Iuchi, N Matsubara, T Ijichi, B Dugas, M Hosokawa

**Affiliations:** 1 Department of Biomolecular Function, Graduate School of Medical Science, Yamagata University, 2-2-2, Iidanishi, Yamagata 990-9585, Japan; 2 Ashama Chemical Co., Tokyo 103-0001, Japan; 3 Research Section of Pathophysiology, Institute for Genetic Medicine, Japan; 4 Oncorex, Hokkaido University, Sapporo 060-0815, Japan; 5 Combi Corp., Saitama 338-0832, Japan; 6 Isocell Nutra SAS, 53 Bld du Général Martial Valin, Paris 75015, France

**Keywords:** orally available superoxide dismutase, metastasis, inflammation-mediated tumour progression, fibrosarcoma cells

## Abstract

Weakly tumorigenic and nonmetastatic QR-32 cells derived from a fibrosarcoma in C57BL6 mouse are converted to malignant cells once they have grown after being coimplanted with a gelatine sponge which induces inflammation. We administered a newly developed peroral superoxide dismutase (SOD), oxykine, and as control vehicle, gliadin and saline, starting 2 days before the coimplantation and continued daily throughout the experiment. In the oxykine group, tumour incidence was lower (41%) than in the gliadin or saline group (83 and 79%, respectively). The inhibitory effect of oxykine was lost when an individual component of oxykine was administered, that is, SOD alone and gliadin alone. The effect was also abolished when administered by intraperitoneal route. When perfused *in situ* with nitroblue tetrazolium, an indicator of superoxide formation, the tumour masses from gliadin and saline groups displayed intense formazan deposition, whereas, those from oxykine group had less deposition. Enzymatic activity of SOD was also increased in oxykine group. Arising tumour cells in gliadin and saline groups acquired metastatic phenotype, but those in oxykine group showed reduced metastatic ability. These results suggested that the orally active SOD derivative prevented tumour progression promoted by inflammation, which is thought to be through scavenging inflammatory cell-derived superoxide anion.

Evidence has been accumulated that many of the cell alterations seen in normal ageing and in various diseases including cancer are due to oxidative damage by active oxygen species (Taniguchi, 1992). Oxygen radicals are a by-product of aerobic respiration and harmful to living cells (Halliwell *et al*, 1992). In tumour tissues, oxygen radicals are generated by cancer cells themselves (Shaughnessy *et al*, 1989; Szatrowski and Nathan, 1991; Oberley and Oberley, 1997), and infiltrating inflammatory cells such as neutrophils (Babior *et al*, 1973; Weissmann *et al*, 1980) are believed to exert tumoricidal effects at their relatively high concentrations. On the other hand, the normal or premalignant cells which are chronically exposed to or escape from a highly cytotoxic concentration of oxygen radicals tend to be transformed to malignant ones or acquire malignant properties as evidenced under certain conditions; namely, oxygen radicals stimulate tumour cell invasion (Shinkai *et al*, 1986) or enhance metastasis (Orr *et al*, 1988).

Most aerobic cells have an enzymatic system to eliminate active oxygen species, because some of these active species are toxic to host. Superoxide dismutases (SODs), catalase, and glutathione peroxidase comprise the major defence system against oxygen toxicity (McCord and Fridovich, 1969). Superoxide dismutases catalyse the dismutation of superoxide anion (O_2_^−^) to produce hydrogen peroxide and O_2_. Superoxide anion is one of the reduced oxygen species generated in cellular metabolism or produced by inflammatory cells at their respiratory burst.

There are three isozymes of SODs in mammalian system (Taniguchi, 1992). Among them, manganese-SOD (manganese-superoxide dismutase (Mn-SOD)) and copper, zinc-SOD (Cu,Zn-SOD) are widely believed to play an important role in carcinogenic processes (Oberley and Buettner, 1979; Dovrat and Gerhon, 1981). It has been found that tumour cells tend to have reduced activities of those SODs compared to normal counterpart (Sykes *et al*, 1978; Oberley and Buettner, 1979; St Clair and Holland, 1991; Brorrello *et al*, 1993), and overexpression of SOD decreases malignant phenotypes in various cancers including breast cancer (Li *et al*, 1995), melanoma (Chruch *et al*, 1993) and glioma (Zhong *et al*, 1997). Moreover, it is known that levels of SODs inversely correlate with metastatic ability of tumour cells (Kwee *et al*, 1991), and that SOD suppresses metastasis of tumour cells *in vivo* (Safford *et al*, 1994; Yoshizaki *et al*, 1994) and inhibits their motility and invasiveness (Muramatsu *et al*, 1995). Superoxide dismutase also acts as differentiation inducer for erythroleukaemia cells (Beckman *et al*, 1989). In other words, deficiency of SOD may disturb redox status in cells, which influences neoplastic transformation and/or the maintenance of the malignant phenotype (Oberley and Buettner, 1979; Loven *et al*, 1984; Oberley and Oberley, 1988). In fact, *in vitro* transformation of normal fibroblast cell lines was carried out by infection with simian virus 40 (SV40) for comparing SOD amounts before and after transformation (Marlhens *et al*, 1985; Oberley *et al*, 1989). The results showed that all of the SV40-transformed cell lines decreased the SOD amount as compared to the original normal fibroblast cell line. Recently, enforced expression of SOD in SV40-transformed cells partially reversed their malignant phenotypes (Yan *et al*, 1996). Also, constitutively expressed high levels of SOD in mouse C3H10T1/2 cells coincided with a decrease in the frequency of radiation-induced neoplastic transformation (St Clair *et al*, 1992). Since an inverse correlation thus exists between its expression and tumour development and progression (Safford *et al*, 1994), it has been hypothesised that SOD is a new tumour suppressor gene (Bravard *et al*, 1992).

Introduction of SODs into tumour cells and/or tumour tissues is theoretically the most efficient strategy for inhibiting both tumour growth and progression. However, for induction of appropriate levels of SOD, it is necessary to use specialised techniques, for example, DNA transfection methods or modification of a specific amino-acid sequence of SOD or modification of SOD to make it stable after injection *in vivo*. It would be beneficial to develop peroral active SOD for wide preclinical usage. In this scheme, Giri and Misra (1984) have reported that, similarly to most proteins, orally administered SOD will be digested in the stomach and only a small portion of SOD will be absorbed in the blood stream through gastrointestinal tract. For minimising the digestion, SOD has been coated with a wheat-based biopolymer, gliadin, (*Triticum vulgare*, Poaceae) which not only prevents gastric digestion (Vouldoukis *et al*, 2003) but also promotes the delivery of the bioactive molecules into the small intestinal mucosa by enhancing the intestinal permeability through activation of a tight-junction-regulating protein, zonulin (Clemente *et al*, 2003).

Our group previously showed that the QR tumour cells obtained from a clonal murine fibrosarcoma were unable to grow in normal syngeneic C57BL/6 mice when injected subcutaneously (2 × 10^5^) whereas they developed tumours after coimplantation with a foreign body, gelatine sponge (Okada *et al*, 1992). We reasoned that the foreign-body-induced inflammation accelerated the progression of QR tumour cells, and suggested the involvement of active oxygen species produced by inflammatory cells in this process (Okada *et al*, 1999; Tazawa *et al*, 2003). The QR-32 tumour cells used in the present study were most sensitive to inflammation-promoted progression, as compared to other QR tumour clones with resistance to progression, since they had significantly decreased Mn-SOD activity (Okada *et al*, 1999). For this reason, we used the tumour cell line for evaluation of the newly developed SOD derivative, oxykine, in prevention of active oxygen species-mediated tumour progression.

We aimed to determine the potential inhibitory effect of oxykine in the model in which we can observe both tumour formation (primary tumour) and progression of tumour cells (acquisition of metastatic phenotype). We herein reported that oxykine reduced primary tumour growth and prevented the acquisition of metastatic property of tumour cells through suppression of superoxide anion at the tumour-growing sites.

## MATERIALS AND METHODS

### Chemicals

Oxykine®, melon-derived SOD and gliadin were provided by Asama Chemical Inc., Tokyo, Japan. The SOD activity of oxykine was 1000 U g^−1^. It was diluted with PBS just before usage and kept on ice until administration. The dose was fixed at 10 mg kg^−1^, and administered intragastrically.

### Tumour cell lines and culture conditions

The origin and characteristics of the tumour cell lines have been described previously (Ishikawa *et al*, 1987). Briefly, BMT-11, a transplantable fibrosarcoma, was induced in a C57BL/6 mouse with 3-methyl-cholanthrene, and a tumorigenic clone BMT-11 cl-9 was subsequently isolated by limiting dilution. BMT-11 cl-9 cells were exposed *in vitro* to quercetin, which gave rise to a number of random subclones (Ishikawa *et al*, 1987). They spontaneously regressed when injected into normal syngeneic mice. The variants were named ‘QR tumour clones’, representing ‘quercetin-induced regressive tumour’. Tumour cells of one of the variant cell clones, QR-32, were used in this study. The culture cell lines established from tumours arisen after coimplantation of QR-32 tumour cells with gelatine sponge in mice were designated as ‘QRsP’, representing ‘progressive tumour variants derived from QR-32 tumour cells co-implanted with gelatine sponge’. The QR-32 tumour cells and QRsP tumour cell lines were maintained in Eagles's minimum essential medium (MEM, Nissui Pharm., Japan) supplemented with 8% fetal bovine serum (Filtron), sodium pyruvate, nonessential amino acids and L-glutamine, at 37°C, in a humidified 5% CO_2_/95% air mixture.

### Mice

Female C57BL/6 mice (5 weeks old) were obtained from Nippon SLC (Hamamatsu, Japan) and used for the experiments. All the mice were maintained in the complete barrier condition, lit from 0700 to 1900, at 23 × 3°C and 50 × 10% humidity, fed with mouse diet (Nihon Nosan Kogyo, Yokohama, Japan) and UV-irradiated water in the germ-free section of Institute for Animal Experimentation, Hokkaido University Graduate School of Medicine. Diet and tap water were available *ad libitum* throughout the experiment.

### Experimental procedures

The experimental protocol was approved by the Committee of Institute for Animal Experimentation, Hokkaido University Graduate School of Medicine (#01139).

The mice at 6 weeks of age were used after 1 week of acclimatisation. A subcutaneous pocket reaching up to the thorax was made from a 10 mm incision on the right flank of the pelvic region in each anaesthetised mouse and one piece of sterile gelatine sponge (10 × 5 × 3 mm^3^ piece; Spongel, Yamanouchi Pharm., Japan) was inserted and the wound was closed with clips. Then QR-32 tumour cells (1 × 10^5^ cells/0.1 ml) were immediately injected into the inserted gelatine sponge (Okada *et al*, 1992).

The mice were divided randomly into three groups and treated with oral administration of oxykine or gliadin at a dose of 10 mg kg^−1^ body weight, or saline. The treatment was carried out every day from 2 days before QR-32 tumour cell implantation to the end of the experiment. Tumour diameter and body weight were measured twice a week during the experiment. All the mice were killed under ether anaesthesia at 28 days after implantation for evaluation of the arising tumours' malignancy and autopsy; simultaneously we removed the subcutaneously growing tumours aseptically to assess whether the arising tumours had acquired malignant phenotype, and used them for establishing individual culture cell lines after mechanical disaggregation with scissors. The detailed procedure has been described elsewhere (Okada *et al*, 1992). The tumour lines were allowed at least four passages in culture to eliminate host cell contamination. Each tumour cell line was injected intravenously (1 × 10^6^ cells) into normal C57BL/6 mice. On day 25, the mice were killed and metastatic nodules at the surface of the lungs or other organs were counted macroscopically.

### Determination of the total number and the types of the cells infiltrated into gelatin sponge

The gelatin sponge pieces subcutaneously injected into the mice treated with oxykine, gliadin, melon-SOD or saline were removed and digested with 0.2% collagenase in serum-free MEM medium for a few minutes at 37°C. After collecting all the infiltrated cells by centrifugation, we counted total number of the cells per piece of gelatin sponge. We also counted differential counts of more than 200 cells in smear preparations of the collected cells stained with May-Gruenwald's and Giemsa solution (Wako Pure Chemical Inc., Osaka, Japan). Mean percentages of differential cells were obtained from the mean values of independent counts by two pathologists.

### Nitroblue tetrazolium (NBT) staining

The mice were killed by cervical dislocation, and the tumour masses were excised and simultaneously stained for a few minutes with NBT (1 mg ml^−1^) in Hank's balanced salt solution (HBSS; 24020-117, Invitrogen, Tokyo, Japan). All unreacted NBT was removed from the tumour masses by washing with HBSS. The NBT-perfused tumour mass was photographed and then fixed with 10% (wt vol^−1^) zinc/formalin for histologic examination of formazan deposits. The procedure followed the method described in a previous paper with slight modifications (Hagen *et al*, 1994).

### Assay for enzymatic activities

The methods for evaluating enzymatic activities have been described previously (Okada *et al*, 1999). Briefly, the tumour tissues were washed with PBS and homogenised in liquid nitrogen. The homogenates were suspended in the PBS and then sonicated on ice four times, for 10 s each, by using a sonicator at intensity of 4 (Microson, Wakenyaku Co. Ltd., Kyoto, Japan). The homogenates were centrifuged at 15 000 rpm for 15 min and the resulting supernatant was used for enzymatic assay. Serum samples were diluted with PBS and used.

Superoxide dismutase activity was measured by the NBT reduction method (Beauchamp and Fridovich, 1971), with slight modifications. Manganese-superoxide dismutase activity was examined at 25°C in 1 ml of 20 mM sodium carbonate buffer, pH 10, containing 0.1 mM EDTA, 0.2 mM xanthine, 12 *μ*M NBT and 1.9 mU xanthine oxidase, and determined from the remaining SOD activity after addition of 2 mM potassium cyanide with a spectrometer at 560 nm. The amount of enzyme-reducing NBT by 50% was defined as one unit of SOD activity. Catalase activity was measured from decomposition of hydrogen peroxide, which was recorded at 230 nm on a chart recorder for 1 min at 36°C. Glutathione peroxidase (GPx) activity was determined by using a *β*-butyl hydroperoxide as substrate. One unit of the enzyme activity was defined as 1 *μ*mol NADPH oxidised min^−1^ at 36°C. The enzyme activities of Mn-SOD, CuZn-SOD, catalase and GPx were expressed as U mg^−1^ protein. Protein concentration was estimated by the Lowry method (Lowry *et al*, 1951).

### Statistical analysis

The significance of the differences in tumour and metastatic incidences was calculated by *χ*^2^ test and the differences in metastatic nodules or body and organ weight were evaluated by Student's *t*-test.

## RESULTS

### Inhibition of growth of QR-32 tumour cells coimplanted with gelatine sponge in syngeneic mice treated with oxykine

Benign fibrosarcoma cells (QR-32) did not develop tumours or form metastasis after subcutaneous (2 × 10^5^ cells) or intravenous (1 × 10^6^ cells) injection into normal syngeneic C57BL/6 mice (Ishikawa *et al*, 1987). Table 1a shows that QR-32 tumour cells coimplanted with a gelatine sponge grew in 15 out of 19 saline-treated mice (79%), 15 out of 18 gliadin-treated mice (83%) and 10 out of 17 oxykine-treated mice (59%). We did not find any significant difference in the tumour incidence among the three groups. However, in an attempt to establish tumour cell lines from the arising tumours, we failed in three out of the 10 tumours arisen in oxykine-treated mice due to scar or necrotic tissues, none of which were viable tumours as far as examined macroscopically and histologically (data not shown). Namely, the number of established cell lines (which is equivalent to the final incidence of tumour formation) was significantly reduced in the oxykine-treated mice compared to those in saline- or gliadin-treated mice (*P*>0.05, Table 1a).

**Figure 1:**
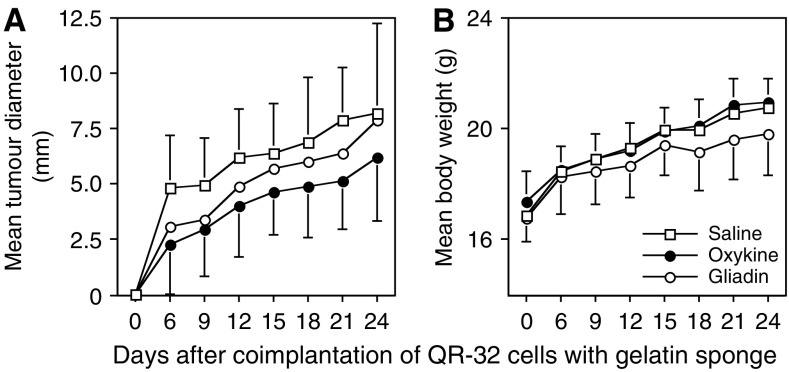
Changes of tumour growth (**A**) and body weight (**B**) following oxykine treatment. Tumour growth curves and body weight curves of normal syngeneic C57BL/6 mice-bearing QR-32 tumour cells coimplanted with a gelatine sponge. Oxykine (closed circle), gliadin (open circle) or saline (open square) had been administered to the mice.

Tumour growth curves of the arising tumours are shown in Figure 1A. Oxykine administration had a slight inhibitory effect on the tumour growth, though it was not significant.

### Inhibition of oxidant production by oxykine treatment at the inflammation-promoted tumour formation

**Figure 2:**
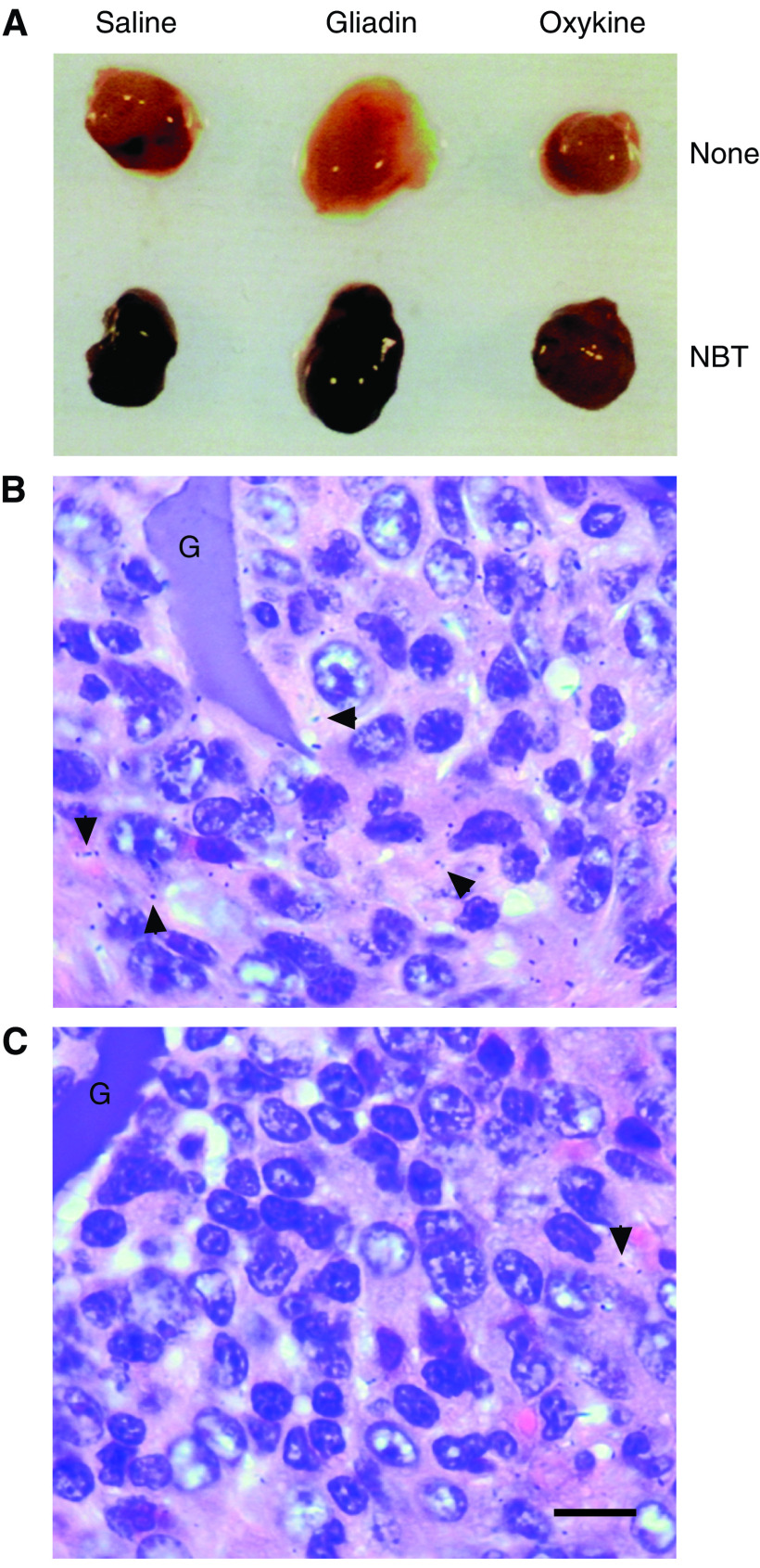
Formazan staining of the tumour tissues from oxykine- and gliadin-treated mice after perfusion with nitroblue tetrazolium (NBT). Tumours were perfused with or without NBT (1 mg ml^−1^), which was followed by wash with Hank's balanced salt solution to remove unreacted NBT (**A**). The tumour tissues were fixed with zinc/formalin and stained with haematoxylin/eosin. Shown is a typical section, indicating intense deposition of formazan crystal (arrow heads). Tumour section obtained from gliadin-treated mice (**B**) and oxykine-treated mice (**C**). G; gelatine sponge filament. Bar; 10 *μ*m.

Nitroblue tetrazolium is a dye that is reduced to an insoluble formazan derivative upon exposure to superoxide (Halliwell and Gutteridge, 1999). The blue-coloured formazan crystal deposition was extensively detectable at the surface of tumour tissues from gliadin- or saline-treated mice. In contrast, tumour tissue from the oxykine-treated mice had less deposit of the crystal (Figure 2A). We performed histological examination and revealed intense deposition of formazan crystal around the gelatine sponge filament, which coincided with the presence of infiltrated inflammatory cells. The density of formazan deposits also reflected the amount of superoxides generated locally. Namely, formazan staining was evident in gliadin-treated tumour tissues (Figure 2B), and, less in oxykine-treated tissues (Figure 2C). These results indicated that tumour tissues in oxykine-treated mice contained lower concentrations of oxygen radicals.

We then examined reactive oxygen species (ROS) statuses in both tumour cells and the inflammatory cells both of which had infiltrated into gelatin sponge. After harvesting those cells by collagenase digestion, we compared cytosolic ROS levels by using an ROS probe, 5-(and-6)-chloromethyl-2′, 7′-dichlorodihydro-fluorescein diacetate (CM-H2DCFDA) and measured intracellular ROS levels by flow cytometry. Mean fluorescence intensities of the cells harvested from the gelatin sponge in the mice orally treated with oxykine, melon-SOD (main component of oxykine), gliadin or saline were 37.4 × 8.6, 42.5 × 13.6, 49.9 × 22.2, 47.5 × 14.2, respectively. We detected positive tendency to decrease the production of ROS in the oxykine-treated group as compared to those in other groups; however, the difference was not significant. We then compared the antioxidative enzyme activities such as those of SOD, glutathione peroxidase and catalase in the serum or tumour tissues after oral administration of oxykine, gliadin or melon-SOD (Table 2). We found that a significant increase in Mn-SOD activity in the tumour tissues from the mice with orally administered oxykine. Since either of the components of oxykine, that is, melon-derived SOD or gliadin, does not have the ability to induce Mn-SOD by itself, the oxykine formulation composed of melon-derived SOD and covered with gliadin was thought necessary to induce preventive effects on the inflammation-induced tumour progression.

### Oral administration is necessary for inhibiting tumour development and acquisition of metastatic phenotype of the QR-32 cells

When we administered oxykine, melon-SOD, gliadin or saline to mice via intraperitoneal route, the effect of oxykine as observed in peroral administration was lost (Table 3). This finding indicated that the inhibition of tumour progression depends on the formulation of the compound and the route of administration. The inhibitory effect of peroral administration of oxykine was also explained by the capacity to have an antioxidative enzyme, SOD, in tumour cells since the oral administration of oxykine-induced SOD in tumour tissues. However, the effect was abolished by switching the administration route to intraperitoneal route (Table 2).

### Inhibition of QRsP/OK tumour lines' acquisition of lung metastatic ability by oxykine

It is the advantage of this model that we can determine whether the arising tumour cells acquire metastatic ability without inflammatory cells, because the tumour cell lines had been established by culturing the cells from tumours arisen in individual mice and were originally their metastatic potential is examined in another normal syngeneic mice (Okada *et al*, 1992). We established *in vitro* culture cell lines from the arising tumours in the mice treated with oxykine, gliadin or saline, and designated them as QRsP/OK, QRsP/GD and QRsP tumour lines, respectively. As Table 1b shows, the lung metastasis incidence was significantly low; only in seven out of 28 lungs had positive metastases developed after i.v. injection of QRsP/OK tumour cell lines, whereas 35 out of 39 lungs and 33 out of 35 lungs had positive metastases after injection of QRsP/GD and QRsP tumour lines, respectively (*P*<0.001). At the time of killing, there was no evidence of typical spontaneous metastasis.

Table 4 shows that the number of colonies per lung and the lung net weight, which indirectly represent metastatic nodules in the lungs. They are significantly less in the mice with QRsP/OK tumour lines than in those with other tumour lines (*P*<0.001). Although there was no reduction of lung metastasis with control vehicle or gliadin treatment, it was suppressed by administration of oxykine (97% inhibition).

### No obvious side effect brought by oxykine treatment

Subcutaneously injected gelatin sponge pieces into the mice with administration of oxykine, gliadin, melon-SOD or saline were removed and the exact number of infiltrated cells was counted per gelatin sponge. Table 5 shows that there was no significant difference among the groups. Then we stained the infiltrated cells and determined their cell types by histological examination, we found that oxykine and other compounds did not make differences in the types of cells infiltrated into gelatin sponge.

The application of oxykine or gliadin (10 mg kg^−1^ day^−1^ for 27 days) did not cause either any obvious side effect such as weight loss, or alteration in the appearance or behaviour of the tumour-bearing mice during the observation period. The data of average body weight are shown in Figure 1B. The values of the oxykine group were not lower than those of the control group (gliadin- or saline treated) throughout the experimental period. There were no significant differences in the final body weight among the treatments (Table 6). Moreover, no significant differences were observed in weights of organs at autopsy either in absolute or ratio to body weight values among the groups (Table 6).

## DISCUSSION

In this study, we showed that an orally available SOD, named oxykine, inhibited inflammation-promoted acquisition of metastatic phenotype of weakly tumorigenic and nonmetastatic murine fibrosarcoma cells without adverse side effect. We also observed suppression of the primary tumour growth by the oxykine treatment.

Several lines of evidence implicate a relationship between induction of SODs in tumour cells and reversion of neoplastic transformation or loss of the malignant phenotype including metastatic property. From these, it has been hypothesised that SOD can be a new tumour suppressor gene (Sykes *et al*, 1978; Bravard *et al*, 1992; Safford *et al*, 1994). It is reported that unbalanced overexpression of SOD protein modulates cellular signal transduction cascades such as tumour –invasion-associated matrix metalloproteases through transactivation of transcription factor(s) (Wenk *et al*, 1999; Nelson *et al*, 2003). Manganese-superoxide dismutase is known to be particularly high in primary hepatoma (Taniguchi, 1992), gastric cancer (Taniguchi, 1992), acute myeloid and/or lymphocytic leukaemias (Nishiura *et al*, 1992), epithelial-type ovarian cancer (Ishikawa *et al*, 1990), central nervous system tumours (Cobbs *et al*, 1996) and neuroblastoma (Kawamura *et al*, 1992); on the other hand, lower SOD levels have generally been demonstrated in other tumour cells and clinical tumour tissues (Oberley and Buettner, 1979; Loven *et al*, 1984; Oberley and Oberley, 1988; Brorrello *et al*, 1993).

To date, eight different techniques have been established to elevate SOD levels in tumour cells or tumour tissues. All the techniques have been reported to reverse malignant phenotypes of tumour cells. They are (i) intravenous or subcutaneous administration of recombinant human SOD which substitutes specific amino acid for stable one (Yoshizaki *et al*, 1994); (ii) intravenous administration of SOD conjugated with a pyran copolymer, for prolongation of its activity (Oda *et al*, 1989); (iii) addition of exogenous liposomal SOD (Beckman *et al*, 1988); (iv) intraperiponeal or subcutaneous administration of a selective SOD mimetic molecule of nonpeptidic and low molecular weight (Samlowski *et al*, 2003); (v) elevation of SOD level by sense cDNA transfection (Safford *et al*, 1994); (vi) inoculation of fibroblasts that are genetically modified to secrete SOD (Tanaka *et al*, 2001); (vii) elevation of SOD levels by exposure to a superoxide generator and subsequent isolation of superoxide-resistant cells (Fernandez-Pol *et al*, 1982); (viii) secondary induction of SOD in tumour tissues by administration of an immunopotentiator which stimulates immune cells to produce SOD-inducible cytokines such as interferon-gamma and tumour necrosis factor-alpha (Habelhah *et al*, 1998). There is no doubt that an orally available SOD would be worth developing for preclinical use of SOD. However, in an experiment using mice, only a small portion (approximately 10%) of orally administered SOD is absorbed through gastrointestinal tract (Giri and Misra, 1984), and most of it was digested, similarly to other proteins, before being absorbed into the blood stream. As a solution of this problem, SOD has been coated with a protective vegetal prolamine (wheat gliadin) layer that not only prevents gastric digestion (Vouldoukis *et al*, 2003) but also promotes the delivery of the bioactive molecule in the mucosa of small intestine (Clemente *et al*, 2003). In this study, we used cantaloupe melon (*Cucumis melo* LC. Cucurbitaceae)-derived SOD. The *C. melo* LC. derived SOD has an SOD activity which is more than five times that of classical melon species, charentais (Vouldoukis *et al*, 2004). In fact, melon, barley plant, broccoli, Brussels sprouts, cabbage, wheat grass and most green plants which we have in daily meals naturally contain large amounts of SOD. Kitagawa *et al* (1986, 1991) reported that X-ray crystallography of plant SOD showed a high structural homology to the mammalian SOD, indicating common characteristics beyond the species barrier such as enzymatic activity (Taniguchi, 1992).

Niitsu and his colleagues have discovered an inhibitory effect of SOD on both experimental and spontaneous pulmonary metastasis in murine models (Yoshizaki *et al*, 1994). They intensively investigated the mechanisms and reported that SOD dramatically suppressed motility and invasion of both human and murine tumour cells (Yoshizaki *et al*, 1994; Muramatsu *et al*, 1995). A similar suppressive effect of SOD on tumour metastasis was observed in the studies of exogenous and endogenous SOD treatments (Kwee *et al*, 1991) and of SOD cDNA transfection (Safford *et al*, 1994). Besides the direct inhibitory effect of SOD on motile phenotype of tumour cells, our present study revealed reduced acquisition of metastatic phenotype in the process of tumour development. We can conclude that the effect we observed was specific to the metastatic ability itself of tumour cells, because we used the culture cell lines established for the evaluation of metastatic ability and the culture condition excluded contamination of oxykine or inflammatory cells. As indicated by *in situ* superoxide production levels in each treatment in Figure 2, we believe that the oxykine administration dismutates superoxides which are produced mainly by gelatine sponge-elicited inflammatory cells and are known as a genotoxic substance to induce gene alterations. From these, we speculate that oxykine may prevent metastasis-associated gene alteration(s) caused by ROS produced by inflammatory cells.

We also observed inhibition of the primary tumour growth in the group with oxykine administration. There are three possible explanations for this. One is that the elevated levels of SOD might lower intracellular levels of O_2_^−^, which in tern downmodulates signal transduction and/or activation of transcription factors to suppress cell growth (Burdon, 1995). Irani *et al* (1997) have recently shown that superoxide acts especially as signal regulator for the stimulation of cell growth through a flavoprotein and Rac1 pathways. Second is that SOD might stimulate tumoricidal immune effector cells. Samlowski *et al* (2003) demonstrated that administration of SOD mimetic nonpeptidic molecule enhanced the cytotoxicity of lymphokine-activated killer (LAK) cells *in vivo*. Indeed, the QR-32 tumour cells are highly sensitive to LAK cells (Okada *et al*, 1994). Third is that oxykine might stimulate immune cells to produce SOD-inducible cytokines and growth factors. Those factors would coordinately synthesise *de novo* SOD at the tumour-growing sites. In our experiments, we revealed that Mn-SOD was induced in tumour cells only by oxykine formulation (Table 2). However, a single component of the oxykine by itself, that is, gliadin alone or SOD alone, does not have ability to induce SOD. At this time, we do not know the precise mechanisms responsible for this, but speculate that the oxykine formulation possibly activates the host immune system besides exerting direct SOD action. Since the main compound of melon-derived SOD induces Th1-dependent immunity (Vouldoukis *et al*, 2003), and the compound covered with gliadin has been identified as a major allergen for wheat-dependent exercise-induced anaphylaxis (Matsuo *et al*, 2005), both components seem to be immunogenic to host. Furthermore, only the oral administration was effective on the induction of SOD and suppression of the tumour progression phenotype (Table 3). Therefore, immunopotentiating effects of orally administered oxykine maybe involved in augmentation of immune system of intestinal tract or whole body through absorption via gastrointestinal tract. We are currently undertaking a study to determine whether oxykine has a role in activation of immune functions.

The formation of human cancer involves a multistage process, that is, initiation, promotion and tumour progression. Oxidative stress is considered to contribute to the whole process from carcinogenesis through induction of mutations or upregulation of cell growth in target cells (Okada, 2002). In this study, we showed inflammation-promoted tumour progression was prevented by administration of a SOD derivative, oxykine. Recent reports have verified that oxykine protects cells from hyperbaric oxygen-induced oxidative stress, that is, breaks of DNA strand in healthy volunteers (Muth *et al*, 2004) and that oxykine prevents oxidative stress-mediated diabetic nephropathy in rodent model of type 2 diabetes (Naito *et al*, 2005). This orally available molecule will be effective in clinical utilisation to prevent both tumour development and progression.

## Figures and Tables

**Table 1:** Inhibition of tumour development and acquisition of metastatic ability of QR-32 tumour cells by administration of oxykine (a) tumorigenicity of QR-32 cells co-implanted with gelatin sponge in mice and (b) characteristics of the arising tumour lines

	**No. of mice with tumour take/no, of mice treated (%)**			
**Treated with^a^**	**Exp. 1**	**Exp. 1**	**Total**	**No. of cell lines establihed/no of tumours tested (%)^b^**
*(a) Tumorigenicity of QR-32 cells co-implanted with gelatin sponge in mice*				
—	—	—	—	—
Saline	8/9 (89)	7/10 (70)	15/19 (79)	15/19^c^ (79)
Gliadin	8/8 (100)	7/10 (70)	15/18 (83)	15/18 (83)
Oxykine	4/7 (57)	6/10 (60)	10/17 (59)	7/17^c^ (41)


	**Lung-colonising ability**		**Other metastasis sites**	
**Cell lines established from the arising tumour**	**Incidence (no. of mice with lung metastasis/no. of mice tested)**	**No. of lung with metastatic nodules**	**Incidence no. of mice with other metastases/no of mice tested)**	**Sites (incidence)**
*(b) Characteristics of the arising tumour lines* d				
QR-32	0/10	0, 0, 0, 0, 0, 0, 0, 0, 0, 0, 0	0/10	None
				
QRsP/-1	3/3	1, 3, 14	0/3	None
QRsP/-2	3/3	8, 13, 20	0/3	None
QRsP/-3	4/4	3, 8, 14, >150	1/4	[O (1/4)]
QRsP/-4	3/4	0, 1, 3, 35	0/4	None
QRsP/-5	4/5	0, 2, 7, 8, 15	1/5	[O (1/5)]
QRsP/-6	4/4	8, 43, >150, >150	2/4	[O (2/4)]
QRsP/-7	4/4	8, 11, >150, >150	2/4	[O (2/4)]
QRsP/-8	4/4	>150, >150, >150, >150	0/4	None
QRsP/-9	4/4	16, 48, 51, 62	0/4	None
				
Total	33/35^e^		6/35	None
				
QRsP/GD-1	4/4	2, 2, 4, 17	0/4	None
QRsP/GD-2	4/4	5, 6, 12, 14	0/4	None
QRsP/GD-3	2/4	0,0,3,8	0/4	None
QRsP/GD-4	3/4	0, 5, 7, 22	1/4	[O (1/4)]
QRsP/GD-5	3/4	0, 3, 6, 12	0/4	None
QRsP/GD-6	4/4	2, 5, 6, 7	1/4	[O (1/4)]
QRsP/GD-7	3/3	16, >150, >150	1/3	[O (1/3), A (1/3)]
QRsP/GD-8	4/4	25, 58, 132, >150	3/4	[O (2/4), LN(1/4]
QRsP/GD-9	4/4	58, 73, >150, >150	0/4	None
QRsP/GD-10	4/4	42, 43, 123, >150	0/4	None
				
Total	35/39		5/39	None
				
QRsP/OK-1	0/4	0, 0, 0, 0	0/4	None
QRsP/OK-2	3/4	0, 2, 3, 18	0/4	None
QRsP/OK-3	2/4	0, 0, 5, 7	0/4	None
QRsP/OK-4	0/4	0, 0, 0, 0	0/4	None
QRsP/OK-5	1/4	0, 0, 0, 8	0/4	None
QRsP/OK-6	0/4	0, 0, 0, 0	0/4	None
QRsP/OK-7	1/4	0, 0, 0, 2	0/4	None
				
Total	7/28^e^		0/28	None

a 1 × 10^5^ QR-32 tumour cells were coimplanted with gelatine sponge in normal mice to which oxykine or gliadin had been administered *per os* (10 mg kg^−1^) once daily throughout the experiment.

b Culture cell lines were separately established from tumours arisen in each mouse.

c *P*<0.05 *vs* saline.

d In a separate experiment, 1 × 10^6^ cells of each cell line were injected into mice. After 25 days, the mice were killed and metastatic nodules at the surface of lung were counted macroscopically. Incidences of lung metastasis were evaluated as follows:

e *P*<0.001 *vs* lung-colonising incidence of saline-treated group.

**Table 2:** Activities of manganese, copper and zinc superoxide dismutases, catalase and glutathione peroxidase in serum or tumour tissues of mice orally or intraperitoneally treated with oxykine, gliadin, melon SOD or saline

		**Antioxidative enzyme activities mg^−1^ protein in**					
		**Tumour tissues**				**Serum**	
**Treatment^a^**	**Route of administration**	**Mn-SOD (U)**	**CuZn-SOD (U)**	**Catalase (U)**	**GPx (mU)**	**Mn-SOD (U)**	**CuZn-SOD (U)**
Saline	Per oral	2.3±1.2^b^	12.9±3.0	36.2±4.0	78.3±12.9	1.7±0.3	14.2±1.4
Gliadin	Per oral	2.7±0.5	15.6±1.7	35.8±11.8	79.5±8.7	1.7±0.5	14.2±0.4
Melon SOD	Per oral	2.9±0.4	14.7±1.1	35.0±12.1	79.8±10.0	1.7±0.5	13.6±2.0
Oxykine	Per oral	5.1±1.1^b^	14.3±3.3	35.8±25.8	77.3±38.2	1.8±0.6	14.7±2.2
							
Saline	Intraperitoneal	2.7±0.7	15.5±2.6	33.3±8.4	83.7±39.0	2.3±1.2	13.9±1.5
Gliadin	Intraperitoneal	2.2±0.8	13.8±2.7	28.5±8.6	66.6±27.0	1.9±0.6	14.9±0.7
Melon SOD	Intraperitoneal	2.9±0.4	14.9±0.7	30.3±6.7	85.5±10.8	1.5±0.3	13.8±0.8
Oxykine	Intraperitoneal	2.5±0.3	15.3±2.9	32.3±12.4	83.3±2.5	2.7±1.2	12.3±2.1

a 1 × 10^5^ QR-32 tumour cells were coimplanted with gelatine sponge in normal mice to which oxykine, gliadin, melon SOD had been administered orally or intraperitoneally (10 mg kg^−1^) every day from 2 days before coimplantation to the end of the experiment. All the mice were killed under ether anaesthesia at 28 days after implantation, and serum and tumour tissues were collected for examination.

b *P*<0.01 *vs* saline group.

**Table 3:** Tumour development and acquisition of metastatic ability of QR-32 tumour cells were inhibited by oral administration of oxykin, but not by a single component of oxykine formulation or by a different administration route

		**Incidence of tumorigenicity**	**Incidence of metastasis**
**Treatment^a^**	**Route of administration**	**No. of mice with tumour take/no. of mice treated (%)**	**No. of mice with lung metastasis/no. of mice treated (%)**
Saline	Per oral	9/10 (90)^b^	20/20 (100)^c^
Gliadin	Per oral	8/9 (89)	18/20 (90)
Melon SOD	Per oral	9/10 (90)	18/20 (90)
Oxykine	Per oral	4/10 (40)^b^	5/19 (26)^c^
			
Saline	Intraperitoneal	10/10 (100)	18/20 (90)
Gliadin	Intraperitoneal	8/10 (80)	19/20 (95)
Melon SOD	Intraperitoneal	10/10 (100)	21/22 (95)
Oxykine	Intraperitoneal	8/10 (80)	20/22 (91)

a 1 × 10^5^ QR-32 tumour cells were coimplanted with gelatine sponge in normal mice to which oxykine, gliadin or melon SOD had been administered orally or intraperitoneally (10 mg kg^−1^) once daily throughout the experiment.

b *P*<0.05 *vs* saline.

c 1 × 10^6^ cells of each cell line established from the arising tumours were injected into mice. After 25 days, the mice were killed and metastatic nodules at the surface of lung were counted macroscopically. Incidences of lung metastasis were evaluated as follows: *P*<0.001 *vs* lung-colonising incidence of saline-treated group.

**Table 4:** Inhibition of QRsP/OK tumour lines' acquisition of metastatic ability by oxykine

**Cell lines**	**Metastatic incidence (%)**	**Lung weight (g)**	**No. of lung metastatic nodules**	**Medium value**	**Range**	**Per cent reduction**
QRsP	33/35 (94)^a^	0.45±0.46^b^	50.1±61.5^a^	12	0–150	0
QRsP/GD	35/39 (90)	0.37±0.39	41.5±55.9	15	0–150	17
QRsP/OK	7/28 (25)^a^	0.18±0.02^b^	1.6±3.9^a^	0	0–18	97

1 × 10^6^ of tumour cells was injected intravenously into C57BL/6 mice. On day 25, the mice were killed and metastatic nodules at the lung surface were counted. Data represent the mean±s.d.

a *P*<0.001.

b *P*<0.005 as compared to QRsP tumour cells.

**Table 5:** Differential leukocyte counts and numbers of cells infiltrated into gelatin sponge in mice with per oral administration of oxykine, gliadin or saline

			**Percentage of differential leukocytes per gelatin sponge-infiltrated cells**				
**Treatment^a^**	**No. of mice examined**	**Total no. of gelatin sponge-infiltrated cells (× 10^5^)**	**Mø/MO**	**PMN**	**LC**	**EOS**	**Others**
Saline	5	15.6±3.1	12.1±6.3	53.6±7.4	28.0±2.8	1.6±0.5	3.1±2.4
Gliadin	6	19.3±5.8	9.9±2.0	49.7±6.0	26.8±2.7	1.6±1.5	5.0±0.8
Melon SOD	6	19.0±5.8	13.1±4.2	50.6±3.1	30.7±2.9	1.4±1.8	4.8±3.7
Oxykine	6	18.4±2.7	8.6±4.2	53.5±5.4	30.8±2.9	1.6±1.1	4.5±2.3

Mø/MO, macrophages/monocytes; PMN, polymorphonuclear neutrophils; LC, lymphocytes; EOS, eosinophils.

a A piece of gelatine sponge was implanted into the subcutaneous space of normal mice to which oxykine, gliadin or melon-SOD had been administered orally (10 mg kg^−1^) once daily for 5 days.

**Table 6:** Averages of final body weights and absolute/relative organ weights of mice treated with oxykine, gliadin or saline administered

			**Liver weight**		**Kidney weight^b^**		**Lung weight**	
**Treatment**	**No. of mice examined**	**Final body weight**	**Absolute (g)**	**Relative (%)^a^**	**Absolute (g)**	**Relative (%)^a^**	**Absolute (g)**	**Relative (%)^a^**
Saline	19	20.6±1.1	1.04±0.19	5.1±0.8	0.29±0.08	1.4±0.4	0.18±0.03	0.9±0.1
Gliadin	18	19.8±1.7	1.00±0.15	5.0±0.8	0.31±0.09	1.6±0.5	0.19±0.05	1.0±0.3
Oxykine	17	20.7±1.4	1.10±0.19	5.4±1.1	0.36±0.10	1.7±0.4	0.22±0.05	1.0±0.2

The mean values of body weight in the groups showed no significant decreases compared with that of the saline-group throughout the experimental period.

a Relative organ weight=organ net weight/body weight (%).

b Combined weight of the two kidneys.
